# Design of Full-Temperature-Range RWGS Catalysts: Impact of Alkali Promoters on Ni/CeO_2_

**DOI:** 10.1021/acs.energyfuels.2c00784

**Published:** 2022-05-25

**Authors:** Jesus Gandara-Loe, Qi Zhang, Juan José Villora-Picó, Antonio Sepúlveda-Escribano, Laura Pastor-Pérez, Tomas Ramirez Reina

**Affiliations:** † Department of Inorganic Chemistry and Materials Sciences Institute, University of Seville-CSIC, Seville 41092, Spain; ‡ Department of Chemical and Process Engineering, University of Surrey, Guildford GU2 7XH, U.K.; § Laboratorio de Materiales Avanzados, Departamento de Química Inorgánica-Instituto Universitario de Materiales de Alicante, Universidad de Alicante, Alicante E-03080, Spain

## Abstract

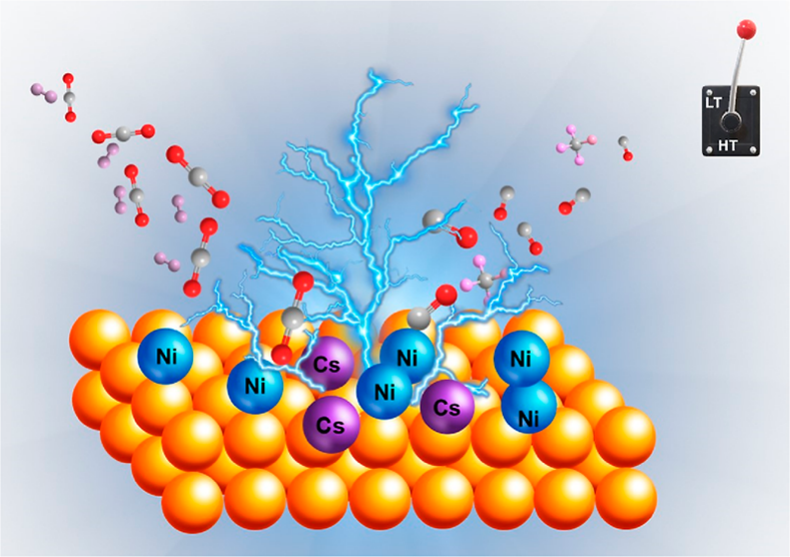

Reverse water gas shift (RWGS) competes with methanation as a direct pathway in the CO_2_ recycling route, with methanation being a dominant process in the low-temperature window and RWGS at higher temperatures. This work showcases the design of multi-component catalysts for a full-temperature-range RWGS behavior by suppressing the methanation reaction at low temperatures. The addition of alkali promoters (Na, K, and Cs) to the reference Ni/CeO_2_ catalyst allows identifying a clear trend in RWGS activation promotion in both low- and high-temperature ranges. Our characterization data evidence changes in the electronic, structural, and textural properties of the reference catalyst when promoted with selected dopants. Such modifications are crucial to displaying an advanced RWGS performance. Among the studied promoters, Cs leads to a more substantial impact on the catalytic activity. Beyond the improved CO selectivity, our best performing catalyst maintains high conversion levels for long-term runs in cyclable temperature ranges, showcasing the versatility of this catalyst for different operating conditions. All in all, this work provides an illustrative example of the impact of promoters on fine-tuning the selectivity of a CO_2_ conversion process, opening new opportunities for CO_2_ utilization strategies enabled by multi-component catalysts.

## 1 Introduction

The rapid increase of world population combined with the development of technological advances powered by uncontrolled fossil fuel exploitation has propitiated the perfect conditions to exponentially accelerate climate change, challenging the future of our planet. For instance, Glasgow COP26, celebrated in 2021, has been a powerful platform to introduce signing state members into future zero-emission technologies, which will contribute to reducing the average global temperature to 2 °C (preferably to 1.5 °C), an essential decrease to prevent climate catastrophes.^1^ More than ever, it is necessary to develop different strategies in the transition to renewable energy that contribute to the reduction of the greenhouse gas effect and mitigate climate changes via CO_2_ capture and utilization. In this context, the conversion of CO_2_ into valuable chemicals such as methane or syngas through catalytic routes has been targeted as potential alternatives for large-scale CO_2_ fixation.^2^

The hydrogenation of exhausted CO_2_ can be used to obtain a synthetic natural gas (CH_4_), which can be further used as a fuel or chemical to produce other high-value products. CO_2_ methanation is an exothermic reaction, thermodynamically favored at lower reaction temperatures between 200 and 400 °C (eq 1).^3^ Nevertheless, this reaction consumes 4 mol of H_2_ per mol CO_2_, making it very challenging unless an abundant source of green H_2_ is available. Alternatively, the reverse water gas shift (RWGS) (eq 2) consumes only one mol of H_2_ per mol of CO_2_, making it more economically appealing in terms of reactant cost. This reaction is mildly endothermic and highly competitive with the exothermic methanation reaction at low–medium temperatures, making the design of catalysts critical to improve the selectivity and the low-temperature activity to either the RWGS reaction or CO_2_ methanation.^4,5^ RWGS is considered a potential route to produce syngas, which is further upgraded in a Fischer–Tropsch (FT) process to obtain fuel hydrocarbon.^6^ Hence, it is essential to design a new generation of advanced catalysts that allows feasible coupling between FT or methanol conversion and RWGS reactions, opening multiple possibilities for sequential conversion of CO_2_ to synthetic fuels and chemicals.
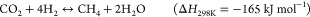
1
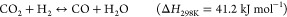
2

In particular, when the RWGS is coupled to a second unit such as FT synthesis or a methanol production reaction, there is a significant temperature gap between both reactors. Typically, the upstream RWGS reaction will run at a high-temperature range around 600–750 °C and downstream unit at 250–400 °C.^7^ Hence, developing low-temperature RWGS with suppressed CO_2_-methanation activity is vital to facilitate overall energy integration.

The CO_2_ molecule is highly stable, which makes the boosting of the kinetic reactions essential through the design of highly active catalysts. For instance, Ni-based catalysts have been the most studied catalyst for CO_2_ hydrogenation not only due to their high catalytic activity but also for their low cost. Also, Ni-based systems have been proven active in RWGS.^8−10^ However, this type of system undergoes deactivation with time due to a variety of reasons, such as agglomeration of Ni particles, carbon deposition on Ni particles, and solid-state reactions involving Ni (i.e., spinel formation).^11^ In this sense, the support has an essential effect on the morphology of the catalyst, adsorption, and catalytic properties that help to prevent the catalyst’s deactivation.^12^ Due to this, the enhancement between the metal and the support is essential to minimize the aforementioned limitations.^13^

Different supports have been employed in Ni-based catalysts such as CeO_2_,^14−17^ ZrO_2_,^18,19^ TiO_2_,^20,21^ SiO_2_,^22,23^ and Al_2_O_3_.^24−26^ Among these supports, CeO_2_ has proved to show exceptional performance in the methanation reaction and RWGS due to its high oxygen storage capacity, oxygen mobility, and high reducibility.^27,28^ For instance, it has been reported that CeO_2_ is able to not only promote the dispersion of Ni particles in Ni–CeO_2_ catalysts but also change Ni properties via metal–support interactions.^29,30^ Recently, different approaches have been suggested to enhance Ni/CeO_2_ catalysts in CO_2_ methanation through the design of nanostructured catalysts. It has been proved that the structuration of catalysts is a route to potentiate the maximum CO_2_ conversion at low temperatures due to the formation of defect sites and oxygen vacancies.^31,32^ Additionally, it has been shown that the addition of small amounts of promoters such as Mg, Co, Ru, Zr, La, Y, and Fe potentiate the stability and the catalytic activity of Ni-based catalysts in the CO_2_ hydrogenation reaction.^33−35^ Recently, le Saché et al. have reported the synthesis of effective switchable ruthenium supported on the CeO_2_–ZrO_2_ catalyst for chemical CO_2_ recycling via CO_2_ methanation and RWGS, which has been observed to be highly active for both reactions just by adjusting the temperature conditions.^36^

Promoters, such as alkali metals (Na and K),^37,38^ alkaline earth metals (Mg, Ca, and Ba),^39,40^ and transition metals (V, Mn, Zn, and Zr),^41−44^ have been probed to enhance the Ni-based catalyst activity due to the change in the chemical surface, which promotes chemical adsorption of CO_2_ and a further decrease in the activation energy of CO_2_ molecules.^45^ For instance, K-promoted Ni-based catalysts have been reported to enhance Ni activity to the RWGS reaction due to the improvement of basicity properties and prevention of carbon deposition.^46,47^ Recently, Azancot et al. have reported an in-depth study to elucidate the catalytic sites formed though the K–Ni interactions and their effect on the dry reforming of methane, which evidenced the formation of Ni–O–K phases promoting the basic sites and improving the stability of the catalyst.^48,49^ However, a high amount of K may cause a decrease in the catalytic activity and possible corrosion issues in the reactor due to K desorption during the reaction.^50^ Similarly, Ni-based catalysts with a low amount of Na have shown to control the selectivity of CO_2_ methanation due to the change in the surface physicochemical properties. An inverse effect was described by Le et al. that showed a negative effect of Na promoted in the Ni/SiO_2_ catalyst due to the change in the intermediates formed during the reaction as a result of basicity modification.^37^ Finally, Cs-promoted Ni-based catalysts have been studied to a lesser extent but mostly related to the RWGS reaction due to its enhancement in suppressing the methanation reaction.^51,52^ Recently, this enhancement of the RWGS reaction has been associated with the effect of Cs in the modification of acid/basic properties of the catalyst, where the alkali metal promotes the formation of basic sites and the stabilization of the active phase, favoring CO selectivity.^53^ Although the addition of alkali metals in catalysts for CO_2_ methanation and RWGS has been explored, a systematic study of the effect of alkali metals in the Ni/CeO_2_ catalyst for low-temperature- and high-temperature RWGS is still lacking.

In this scenario, the present paper showcases the design of multi-component alkali-metal-promoted Ni/CeO_2_ catalysts with switchable activity for both high- and low-temperature RWGS challenging the competition of CO_2_ methanation. Along with the design of the catalyst, our work aims to elucidate the effect of the selected promoters in each reaction to establish activity–structure correlations, opening new routes for rational catalyst design, which are urgently needed to address global warming.

## 2 Techniques and Experimental Methodologies

### 2.1 Catalyst Synthesis

The alkali-promoted Ni/CeO_2_ catalysts were synthesized by the wet co-impregnation method, as described elsewhere. For the nonpromoted catalyst (Ni/CeO_2_), 1.5 g of Ni(NO_3_)_2_·6H_2_O (5.11 mmol) was dissolved in 30 mL of water and mixed with 2.7 g of CeO_2_ (commercialized by Rhone-Poulenc) while maintaining in homogeneous impregnation for 4 h at room temperature. After the impregnation, the water from the soaked powder was slowly removed by a vacuum-assisted rotary evaporator. The material was dried in air at 100 °C overnight prior to the calcination at 550 °C at a rate of 10 °C/min and kept for 4 h. The nickel content in the Ni/CeO_2_ catalyst was calculated to be 10 wt %. Similarly, 3 g of the alkali metal-promoted catalyst x–Ni/CeO_2_ (x = K, Na, Cs) were prepared by mixing 1.5 g of Ni(NO_3_)_2_·6H_2_O (5.11 mmol) and xNO_3_ (to maintain a nickel content of 10 wt % and a x/Ni molar ratio of 1:10) (0.511 mmol) and were dissolved in 30 mL of water and mixed with CeO_2_. After the impregnation for 4 h, the water was evacuated from the material, and the catalysts were dried at 100 °C overnight, previous to the calcination at 550 °C for 4 h.

### 2.2 X-ray Diffraction Measurements

X-ray diffraction measurements were performed to evaluate the crystallinity and phase identification of the catalyst using diffractometer equipment (Siemens D-500) equipped with a Ni-filtered Cu Kα radiation source (40 mA, 45 kV) between 2θ = 10–80°. The patterns were collected using a step size of 0.05° and a step time of 300 s.

### 2.3 N_2_ Adsorption Isotherms at −196 °C

A volumetric equipment brand Micromeritics model ASAP 2010 instrument was used to evaluate the textural properties of the materials through the N_2_ adsorption–desorption isotherms at −196 °C. The samples were degassed at 150 °C overnight in vacuum conditions before the measurements. The Brunauer–Emmett–Teller (BET) method was applied to the experimental data to determine the specific surface area, while the Barrett–Joyner–Halenda (BJH) method was used to obtain the average pore size distribution and pore volume.

### 2.4 Scanning Electron Microscopy

The catalysts were morphologically evaluated using a Hitachi S4800 microscope equipped with a cold cathode field-emission gun (voltage, from 0.5 to 30 kV), resolution of 1 nm (at 15 kV) in order to elucidate the metal dispersion and the morphology of the synthesized catalysts. Additionally, the microscope is equipped with a Bruker-X Flash-4010 EDX detector with a resolution of 133 eV (at the Mn Kα line).

### 2.5 X-ray Photoelectron Spectroscopy

X-ray photoelectron spectroscopy (XPS) spectra of the studied catalyst were collected using a constant energy mode K α spectrometer Thermo Fisher Scientific. This equipment poses an energy scan of 200 eV (survey) and a narrow scan of 50 eV aiming to measure the whole energy band and selectively measure the targeted elements. All XPS spectra were collected using an Al Kα radiation source of 1486.6 eV, with a monochromator crystal at 3 mA × 12 kV. The reference binding energy was established using the C 1s core level, which is located at 284.6 eV binding energy (BE). All catalysts were reduced ex situ at 350 °C and conserved in octane until analysis. A residual pressure of ca., 5 × 10^–7^ N/m^2^, was established as a set point in the analysis chamber prior to the experiments.

### 2.6 Hydrogen Temperature-Programmed Reduction

Hydrogen temperature-programmed reduction (H_2_-TPR) measurements were conducted in a conventional U-shape reactor. In a typical experiment, 50 mg of the catalyst was exposed to a gaseous mixture of 5% H_2_/Ar, and a flow rate of 50 mL/min from room temperature to 900 °C with a heating rate of 10 °C/min was used. A thermal conductivity detector was used to track the H_2_ consumption. Prior to the TPR measurements, the catalyst was pre-treated with He (50 mL/min) at 150 °C for 1 h to eliminate possible impurities.

### 2.7 Catalytic Activity Test

The catalytic activity of the materials was evaluated using a continuous flow quartz fixed-bed reactor setup with an outside diameter of 0.5 in., which was placed vertically in a tubular furnace. The catalysts were located on top of a quartz wool bed inside that reactor. A thermocouple was located at the center of the reactor to monitor the temperature throughout the experiments. Typically, 250 mg of sample (100–200 μm particle size) was diluted in SiC to obtain a 1 cm^3^ reactor bed to avoid heat-transfer limitations and placed into a cylindrical reactor. Prior to the reaction, the sample was reduced and activated using a flow of 50 mL/min of H_2_/N_2_ (50/50%) at 800 °C for 1 h (step skipped for the nonreduced catalysts). After the reduction of the catalyst, a 50 mL/min gas mixture flow (50% N_2_, 40% H_2_, and 10% CO_2_) was set in the inlet of the reactor and evaluated in the temperature range from 200 to 800 °C, using a temperature increasing rate of 10 °C/min and keeping the system in isothermal conditions in each step for 1 h. The weight hourly space velocity (WHSV) was constantly kept at 12,000 mL g^–1^ h^–1^ with a H_2_/CO_2_ ratio of 4:1. The gases were fed using Aalborg GFC17S-VBL6-AO mass flow controllers with an accuracy of ±1%. Prior to the analysis of the outlet stream, the produced water was condensed using a chiller-condenser system. The outlet stream was quantified using an ABB AO2020 Advanced Optima Process Gas Analyzer, where several values are extracted during the 1 h isothermal condition step. The catalytic activity was quantified calculating the CO_2_ conversion (eq 3) and CH_4_ (eq 4) and CO (eq 5) selectivities
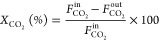
3
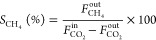
4
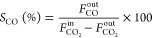
5where  is the concentration of CO_2_ in the inlet stream and , , and *F*_CO_^out^ correspond to the concentrations of CO_2_, CH_4_, and CO at the outlet streams, respectively.

ChemStations’ ChemCad software (Soave–Redlich–Kwong equation of state) was used to obtain the theoretical thermodynamic equilibrium for CO_2_ conversion in the analysis temperature range and flow conditions aiming to provide a complete overview of the experimental results. The resulting theoretical equilibrium curve is shown in the catalyst CO_2_ conversion performance plot figures.

### 2.8 Catalyst Stability Test

The stability of the catalysts was evaluated by performing switching cycles in the temperature range of low- (CO_2_ methanation range) and high-temperature RWGS using the same reaction conditions as used in catalyst activity tests.

## 3 Results

### 3.1 X-ray Diffraction

**Figure 1:**
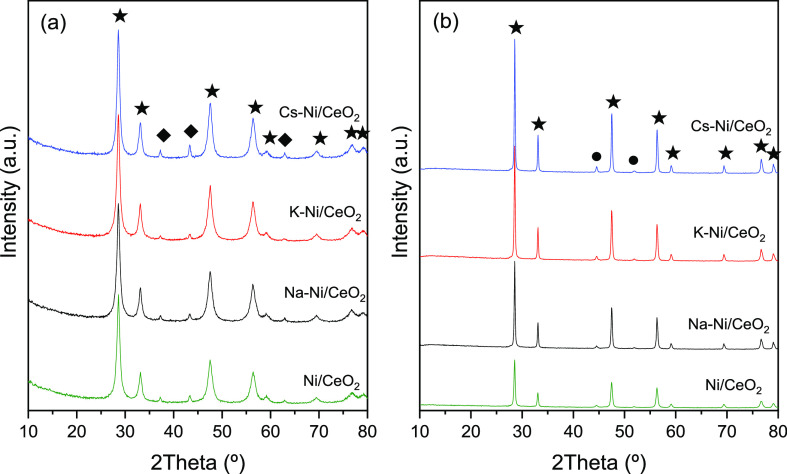
XRD diffraction patterns of (a) calcined catalysts and (b) H_2_ reduced catalysts. [(★) CeO_2_ JDPS 34-0394, (●) Ni JDPS 04-0850, and (◆) NiO JDPS 47-1094].

X-ray diffraction (XRD) measurements were used to evaluate the structural characteristics of the catalysts, as depicted in Figure 1. The XRD patterns of the fresh calcined catalysts (Figure 1a) show the typical peaks of the CeO_2_ at 2θ (°) = 28.67, 33.05, 44.55, 56.50, 59.26, 69.41, 76.63, and 78.91. Additionally, reflection planes attributed to the NiO are observed at ca. 2θ (°) = 37.14, 43.1, and 62.82 (JDPS 47-1094). However, after the reduction under a H_2_ atmosphere, higher crystallinity and bigger particles of CeO_2_ are observed (Figure 1b). In addition, the reflection planes (111) and (200) of metallic Ni at 2θ = 44.7 and 51.9, respectively, appear upon treatment under a reductive atmosphere. The absence of any diffraction peak attributed to the alkali promoters in the calcined and reduced catalyst is attributed to the small amount of metal (1:10 x/Ni molar ratio x = Na, K, and Cs) and also to their high dispersion. The average crystal size was quantified by using the Scherrer equation evaluating the FMWH as summarized in Table 1. The Ni crystal size in both the calcined and reduced catalysts increases with the addition of the promoters. However, the increase in the Ni particle size is more evident in the reduced catalyst. For instance, Chen et al. showed that K species in Ni-based catalysts weakens the Ni^2+^ support interactions, enhancing the particle growth.^47,50^ Similarly, average crystal sizes for CeO_2_ were calculated using the (111) diffraction plane at 2θ = 28.6° (Table 1), where an increase is observed in the CeO_2_ crystal size after the incorporation of K, Na, and Cs in the catalyst. This increase can be explained due to the modifications in grain boundary mobility brought by the promoter loading. For instance, it has been reported that at low Na^+^ loadings (0.5–2 wt %), Na^+^ cations diffuse and incorporate into the CeO_2_ lattice, generating oxygen vacancies that enhance the grain boundary mobility.^54,55^

**Table 1:** Crystal Size and Textural Properties of the Calcined and Reduced Catalysts

	crystal size (nm)					
	calcined		reduced			
catalyst	NiO^a^	CeO_2_^c^	Ni^b^	CeO_2_^c^	*S*_BET_(m^2^/g)	*V*_T_ (cm^3^/g)
Ni/CeO_2_	17.6	16.4	18.9	27.9	*132*	*0.061*
Na–Ni/CeO_2_	20.4	21.2	37.9	34.9	*113*	*0.051*
K–Ni/CeO_2_	17.5	18.5	28.3	34.9	*106*	*0.046*
Cs–Ni/CeO_2_	20.5	21.2	28.3	34.9	*101*	*0.042*

a Calculated using the NiO(200) diffraction plane.

b Calculated using the Ni^0^(111) diffraction plane.

c Calculated using the CeO_2_(111) diffraction plane.

### 3.2 Nitrogen Isotherms at −196 °C

**Figure 2:**
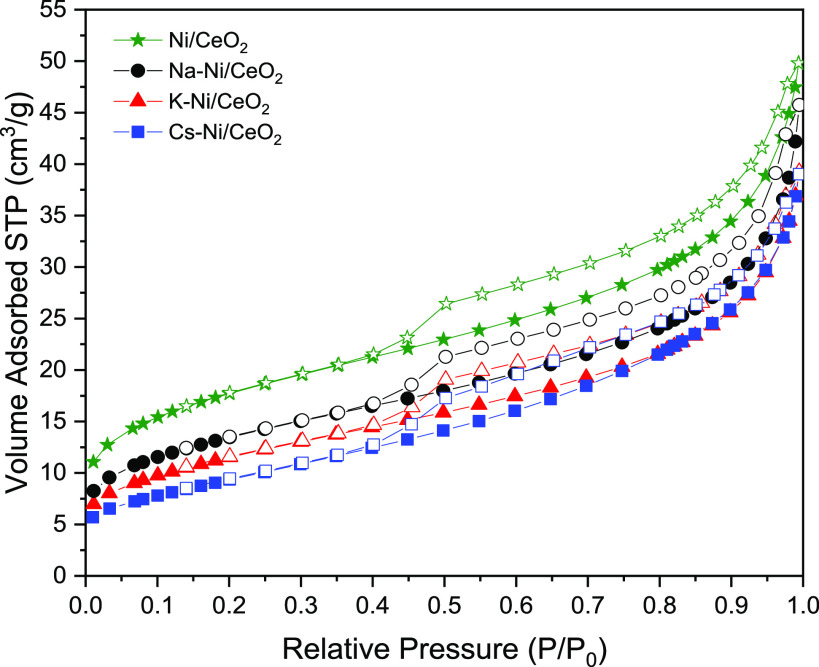
N_2_ isotherms at −196 °C of the evaluated catalysts.

Nitrogen adsorption/desorption isotherms were recorded at −196 °C to evaluate the textural properties of the catalysts. As observed in Figure 2, the four catalysts present a typical isotherm of mesoporous materials. Furthermore, all samples show a type IV isotherm which is characteristic of mesoporous materials with a hysteresis loop H3.^56^ The surface area of the alkali-promoted catalysts suffers from not only a notable decrease in comparison with the Ni/CeO_2_ catalyst due to the partial block of the cavities but also the formation of bigger particles in the surface of the catalyst, which are in agreement with the tendency observed in the crystal size. In addition, the pore volume of the promoted catalyst shows a reduction of ca. 23% as a result of the incorporation of the promoters. This decrease in the textural properties follows the trend of Na < K < Cs, which is concordant with the promoter atomic radii (i.e., Na, 180 pm; K, 220 pm; and Cs, 265 pm). Finally, Figure S1 shows the pore size distribution of the catalysts, where it is observed that the incorporation of the promoters do not have a considerable effect in the pore characteristics compared to the monometallic catalyst.

### 3.3 Scanning Electron Microscopy

**Figure 3:**
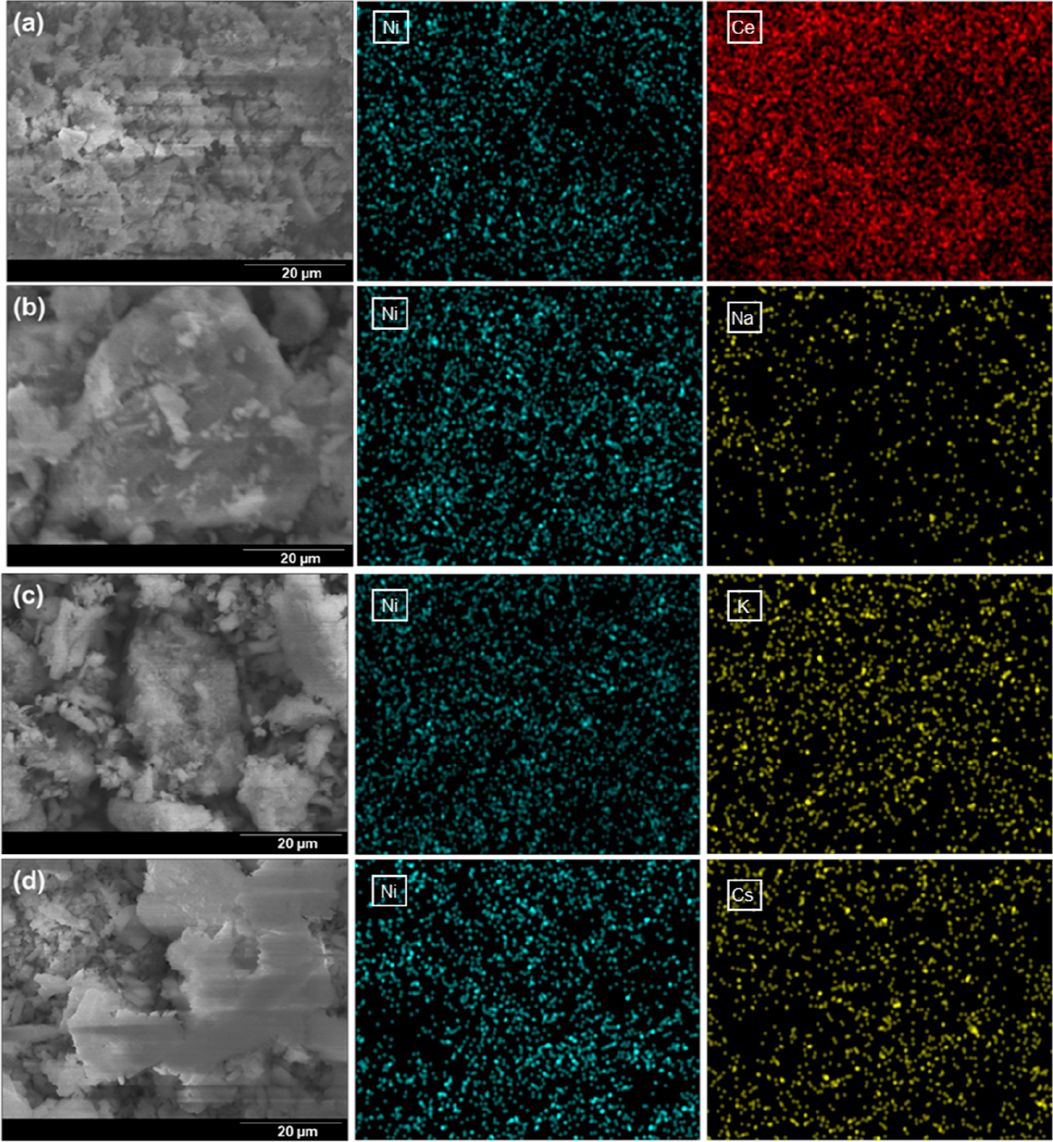
SEM images and EDS spectra of (a) Ni/CeO_2_, (b) Na–Ni/CeO_2_, (c) K–Ni/CeO_2_, and (d) Cs–Ni/CeO_2_.

The elemental composition and morphology of the alkali-promoted Ni supported in CeO_2_ catalysts were studied using a scanning electron microscope, equipped with an energy-dispersive X-ray spectrometer. Figure 3 shows the scanning electron microscopy–energy-dispersive X-ray spectrometry (SEM–EDS) images of the catalysts. In all catalysts, a Ni phase homogeneously dispersed through the support is observed. Furthermore, in the case of Na-, K-, and Cs-promoted Ni-based catalysts, the alkali metals are also found to be highly dispersed on the surface of the catalyst in close contact with Ni particles favoring active phase–promoter interactions. From the morphology perspective, no significant differences among the studied samples are observed.

### 3.4 X-ray Photoelectron Spectroscopy

#### 3.4.1 Calcined Samples

**Figure 4:**
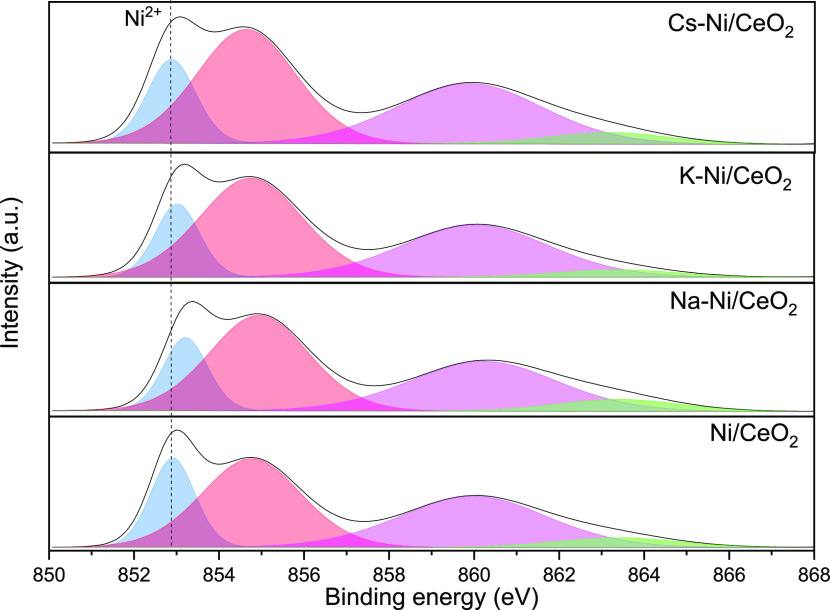
XPS spectra of the Ni 2p_3/2_ region for all the calcined samples.

X-ray photoelectron spectroscopy (XPS) analysis was carried out to elucidate the effect of promoters on the chemical environment states of Ni and surface composition. Figure 4 summarizes the Ni 2p_3/2_ XPS spectra of the calcined samples, and the relative distribution of Ni^2+^ in the CeO_2_ support is summarized in Table 2. Typical peaks of Ni^2+^ in different environments appear at BEs close to 852.9 and 855 eV. However, in alkali-promoted catalysts, these peaks shifted to higher BE values as a consequence of the change of the environmental surface chemistry caused by the addition of the promoters, as previously reported elsewhere.^47,54^ For instance, previous works showcased the partial formation of K_2_NiO_2_ or K_2_NiO_3_ phases after sample calcination at 450 °C.^57^ As reported in Table 2, the Ni^2+^/CeO_2_ ratio suffered a decrease after the incorporation of the promoters, evidencing the partial coverage of Ni and a possible particle size increase due to the weakening of the Ni^2+^–support interaction as discussed in the X-ray Diffraction section.

**Table 2:** BEs of the Ni 2p_3/2_ Levels for the Calcined Catalysts and Ni^2+^/CeO_2_ Atomic Ratios

	Ni 2p_3/2_	
	Ni^2+^	Ni^2+^/CeO_2_
catalysts	BE (eV)	a.u.
Ni/CeO_2_	852.9–854.7	1.84
Na–Ni/CeO_2_	853.2–854.9	0.77
K–Ni/CeO_2_	853.0–854.7	0.58
Cs–Ni/CeO_2_	852.8–854.6	0.58

#### 3.4.2 Reduced Samples

**Figure 5:**
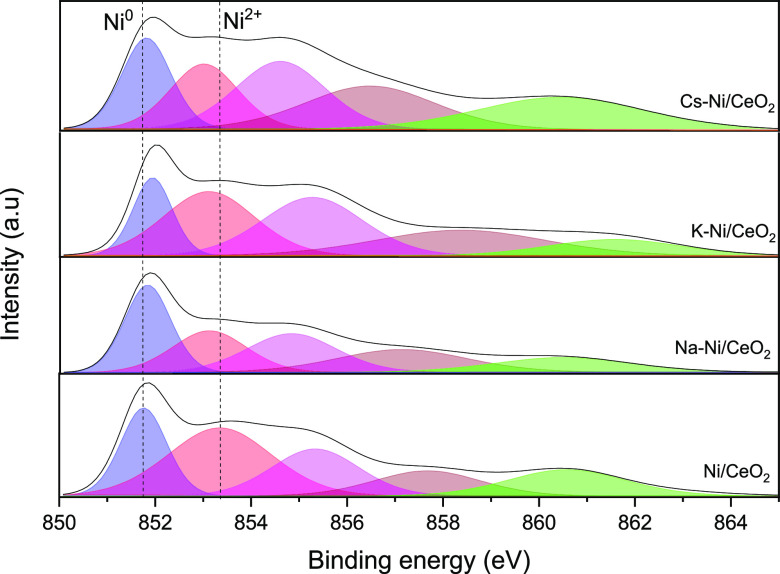
XPS spectra of the Ni 2p_3/2_ region for all the reduced samples.

On the other hand, the XPS spectra of the H_2_ pre-reduced samples present several differences in comparison with that of the nonactivated catalysts. As summarized in Table 3, the Ni^0^/(Ni_total_) ratio increases after the incorporation of the promoters, which reveals that the addition of alkali metals facilitates the reduction of Ni in the x-Ni/CeO_2_ (x = Na and Cs) catalysts, showing a promoting effect of alkali metals in the reducibility behavior of NiO particles as well as a promotional effect rendering higher surface metallic Ni availability. Interestingly, the opposite was observed for the K-containing sample. This could be attributed to the formation of K_2_NiO_2_ or K_2_NiO_3_ phases that make the Ni reduction difficult. Additionally, in the presence of Ni^0^, after the reduction of catalysts, it is important to mention that in the case of promoted catalysts, the binding energy of Ni^2+^ species is shifted to a lower BE (Figure 5), which may also be an indicator of the electron transference between the alkali metal and Ni, as has been described in previous publications.^58^ Particularly, the observed shift of BEs of Ni^0^ and Ni^2+^ in the XPS spectra of Cs-promoted catalysts becomes more evident due to the marked electropositive nature of Cs, which presents a high tendency to transfer electrons and thus leads to remarkable alteration of the surface chemical environment in the Cs-containing system.

**Table 3:** BEs of the Ni 2p_3/2_ Levels for the Reduced Catalysts and Ni^0^/Ni_total_ Atomic Ratios

	Ni 2p_3/2_			Ce 3d3/2
	Ni^0^	Ni^2+^	Ni^0^/(Ni_total_)	Ce^4+^
catalysts	BE (eV)	BE (eV)	%	BE (eV)
Ni/CeO_2_	851.8	853.4–855.3	26.6	915.94
Na–Ni/CeO_2_	851.8	853.1–854.8	38.1	916.11
K–Ni/CeO_2_	851.9	853.1–855.2	20.6	916.09
Cs–Ni/CeO_2_	851.8	853.0–854.6	29.9	916.13

### 3.5 H_2_-Temperature-Programmed Reduction

**Figure 6:**
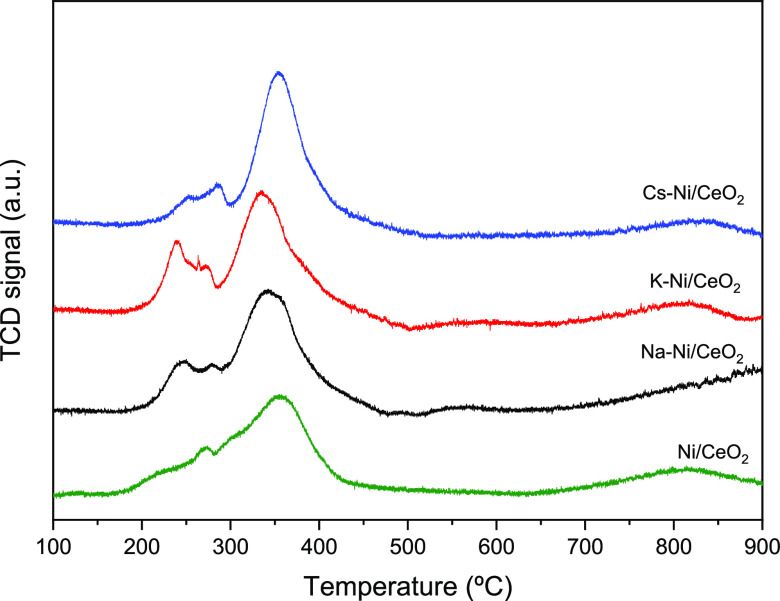
H_2_-TPR profiles of all the studied catalysts.

Further understanding of the redox properties, as well as the metal–support and metal–promoter interaction, was gained from TPR measurements. Figure 6 shows the H_2_-TPR consumption profiles of the Ni-based catalyst and its alkali-promoted counterparts. All samples show the characteristic peak centered at ca. 350 °C, which is commonly assigned to the reduction of Ni^2+^ interacting with the CeO_2_ support and the reduction of surface ceria species that can be easily reduced by dissociated hydrogen on metallic Ni.^59,60^ The TPR peak above 500 °C is attributed to the reduction of bulk ceria. Additionally, for the Ni/CeO_2_ samples, a smaller peak can be seen at lower temperatures (ca. 250 °C), which can be attributed to the reduction of small NiO particles and NiO particles weakly interacting with ceria.^61^ Interestingly, promoted catalysts present an increase in H_2_ consumption at this lower-temperature zone. For the three promoter samples, two small reduction events appear, which testifies the boosting effect of the addition of the promoters in the reducibility as a result of the incorporation or close contact of alkali cations with the CeO_2_ lattice, resulting in lattice distortion and creation of oxygen vacancies, as it has been suggested in previous publications.^54^ This distortion and generation of oxygen may increase the oxygen mobility and, as a consequence, enhance the reducibility of the NiO particles.^62^ Furthermore, this reducibility can be equally associated with the promoter–nickel interactions that allow a promoted reduction of Ni due to the nature of alkali metals in good agreement with our obtained XPS data. It can be highlighted that the K–Ni/CeO_2_ sample presents a slight shift to lower temperatures of the entire TPR profile that allows us to correlate these results with those obtained by XRD. It is well known that the reduction temperature is directly correlated with the metal particle size, which is demonstrated for this catalyst as a smaller crystallite size of NiO and CeO_2_ (Table 1) as the reason for the small change in the reduction temperature.

### 3.6 Catalytic Activity

Given the multiple scenarios and flue gases in which CO_2_ conversion units may fit, we envisage versatile catalysts as key players for industrial decarbonization. In this sense, we tested our catalysts’ RWGS in the full temperature range to allow end-product flexibility. For instance, the low-temperature range pursues the integration of RWGS with an FT unit to produce synthetic fuels, while the high-temperature RWGS leads to CO (and syngas) as a valuable feedstock for the chemical industry. Catalysts were pre-conditioned (reduction) with H_2_ before the reaction took place. However, the pre-activation carries an extra cost to the process, and because both RWGS and CO_2_ methanation are predominantly reductive atmospheres, fresh (nonreduced) samples were also tested to check whether the pre-conditioning step could be ruled out.

#### 3.6.1 Nonreduced and Reduced Catalysts

**Figure 7:**
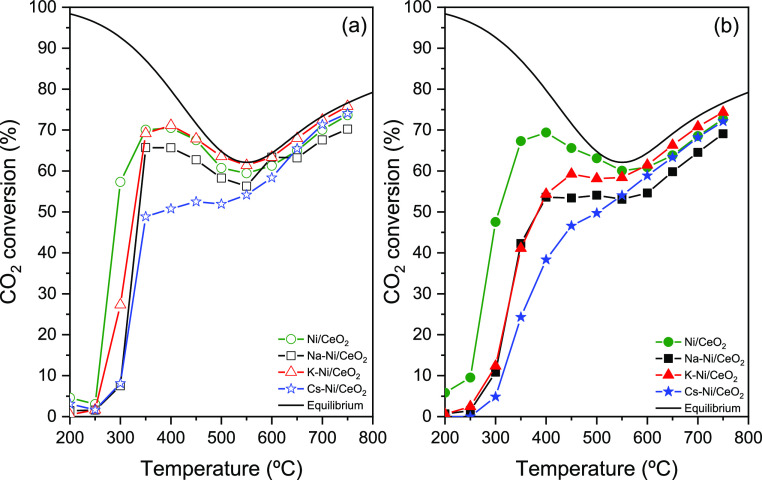
CO_2_ conversion of (a) nonreduced and (b) pre-reduced at 750 °C catalysts. Conditions: atmospheric pressure, WHSV = 12,000 mL g_cat_^–1^ h^–1^, H_2_/CO_2_ = 4:1.

Figure 7a,b shows the CO_2_ conversion of Ni/CeO_2_ and Na-, K-, and Cs-promoted catalysts without pre-activation treatment and H_2_ reduced ones, respectively. The 1:10 promoter/Ni ratio in the catalyst was selected based on previous publications that suggested that low concentrations of alkali metals can help to not only enhance and control the selectivity in the methanation reaction and RGWS via alkali metal–Ni interactions but also generate oxygen vacancies in CeO_2_ due to incorporation of alkali in the oxide lattice.^54^ As observed in Figure 7a, the addition of Na, K, and Cs has an effect on the CO_2_ conversion with a reduction of the catalytic activity following the trend Cs < Na < K at lower temperatures. This is in agreement with the catalyst’s characterization, where a partial coverage of Ni particles by promoters is observed. The decrease in the catalytic activity may be attributed to this partial coverage of Ni nanoparticles by the promoters, obtaining the lower conversion with the catalysts promoted with the bigger alkali metals (Cs, 2.67 Å; K, 2.35 Å; and Na, 1.90 Å atomic radius). In the case of Na-promoted catalysts, this decrease in the catalytic activity is also affected by the bigger particle size. As it was observed in the XRD, the Na-promoted catalyst presents a bigger particle size compared with other catalysts from the series. This inherently causes the worst active phase dispersion and fewer active site availability and, by consequence, a decrease in the catalytic activity. Additionally, after performing a reduction treatment of the catalysts in a H_2_ atmosphere up to 750 °C (Figure 7b), this decrease in the catalytic activity becomes more evident following a similar trend between the alkali metals. This trend may be attributed to not only the effect of the promoters on the particle size and dispersion of the active phase of the catalysts but also the thereof mentioned partial coverage of some Ni active sites by the alkali metals.^37,63^

**Figure 8:**
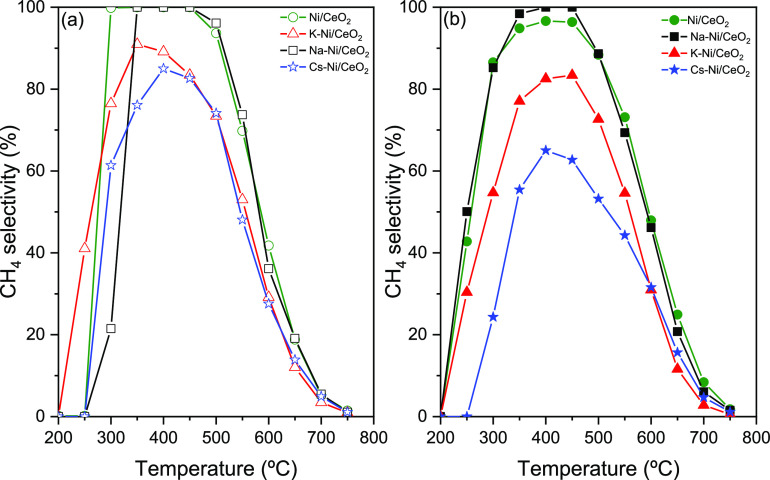
CH_4_ selectivity for (a) nonreduced and (b) reduced at 750 °C catalysts. Conditions: atmospheric pressure, WHSV = 12,000 mL g_cat_^–1^ h^–1^, H_2_/CO_2_ = 4:1.

**Figure 9:**
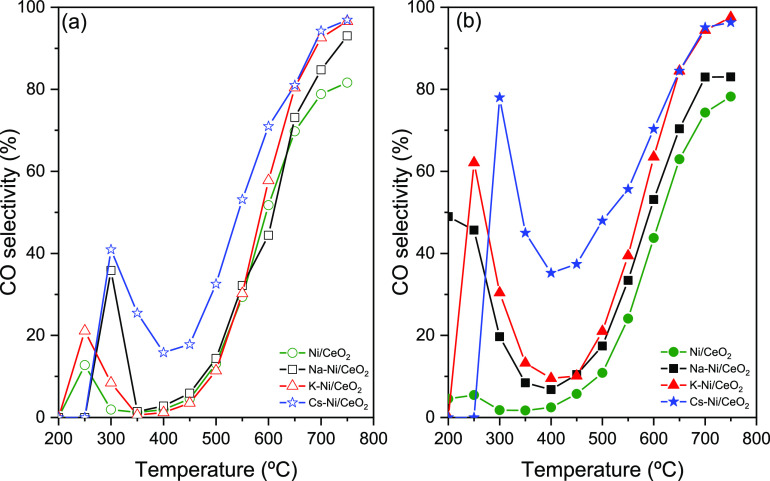
CO selectivity for (a) nonreduced and (b) reduced at 750 °C catalysts. Conditions: atmospheric pressure, WHSV = 12,000 mL g_cat_^–1^ h^–1^, H_2_/CO_2_ = 4:1.

Similarly, if we observe Figure 8a,b, which presents the CH_4_ selectivity in the nonactivated and in the reduced catalysts, respectively, the promoted catalysts show a different performance in comparison to the reference system, especially in reduced catalysts where we observed a more notorious suppressive effect of the methanation reaction in the promoted catalysts. For instance, Zhang et al. suggested that the addition of alkali metals with an alkaline support in Ni-based catalysts may stir the reaction by the generation of HCOO* and CO_3_^2–^ intermediates, which slow down their further reduction to CH_4_.^64^ The opposite is observed in the CO_2_ conversion, and the addition of the alkali metals has a considerable promoting effect on the increase of CO selectivity (Figure 9a,b). Despite the promoting effect of the three alkali metals in CO selectivity, in the specific case of the Cs–Ni/CeO_2_ catalyst, an important improvement is observed in the selectivity in both low- and high-temperature ranges, which confirms the effect of the promoter in the performance of low- and high-temperature RWGS. Most importantly, even though Cs slightly lowers CO_2_ conversion in the low-temperature range, the selectivity for RWGS is remarkably boosted. When we focus on CH_4_ selectivity in Figure 8b, we can find that at low temperatures the Cs–Ni/CeO_2_ catalyst is able to suppress the CH_4_ formation by approximately 30%, while the CO selectivity (Figure 9b) is enhanced, indicating the suitability of this multicomponent system for all temperature range RWGS.

As observed in Figure 9a,b, this CO-selective boosting effect and CH_4_ suppression at low temperatures are less evident in the Na–Ni/CeO_2_ catalyst, which may be attributed to the bigger particle size and low convergence of the active sites compared to other catalysts. Additionally, in general terms, this suppression of CH_4_ and boosting of CO formation can also be explained as due to the lower hydrogen coverage of the active metals and the hindrance the H-assisted CO dissociation.^65,66^

A similar phenomenon is observed in the K-promoted Ni/CeO_2_ catalysts, while in the nonreduced catalyst, the effect of the alkali metal is less drastic in terms of suppressing the CH_4_ selectivity and enhancing the CO formation. The H_2_-reduced catalysts present a decrease in the catalytic activity similar to that observed in Na–Ni/CeO_2_. Despite the decrease in CO_2_ conversion in the K–Ni/CeO_2_ catalyst (Figure 7), there is a clear effect of K in the suppressing effect of CH_4_ formation (Figure 8), while boosting the formation of CO at low temperatures (Figure 9), reaching a CO selectivity up to 60% at 250 °C. The boosting of CO formation in K-promoted catalysts can be explained by the weak interactions of CO and the catalyst surface, which may cause the CO species to desorb rather than be further hydrogenated into CH_4_.^67^ Additionally, the reduction in the CO_2_ conversion at low temperatures may be explained due to the interactions K–Ni that covers Ni nanoparticles that also favor weaker surface–CO interactions, as suggested in previous publications.^50^

Overall, the CO boosting effect of the promoted catalysts compared with that of the nonpromoted Ni/CeO_2_ catalyst gives evidence of the suitability of these catalyst systems in the design of novel catalysts being able to work in the complete temperature range of the RWGS reaction. For instance, from Table 4 that summarizes the CO production rates in the evaluated catalysts at a low temperature (350 °C), it can be observed that using the Cs-promoted catalyst it is possible to obtain a CO production rate that is 10 times bigger compared to the nonpromoted catalyst in confirming our strategy of designing a full-temperature-range RWGS catalyst. Furthermore, all alkali-promoted catalysts show an improvement in the CO production compared to that of the monometallic Ni-based catalyst.

**Table 4:** Comparative CO Production Rates (μmol CO•g_cat._^–1^•s^–1^) of the Studied Catalysts

	CO rate (μmol CO g_cat._^–1^ s^–1^)			
sample	nonreduced	reduced	temperature (°C)	reference
Ni/CeO_2_	0.126	0.169	350	this work
K–Ni/CeO_2_	0.062	0.813	350	this work
Na–Ni/CeO_2_	0.136	0.529	350	this work
Cs–Ni/CeO_2_	0.882	1.425	350	this work
Ni–TiO_2_		1.767	360	Li et al.^68^
Ni–TiO_2_–NH_3_		0.411	360	Li et al.^68^
β-Mo_2_C		1.000	300	Zhang et al.^69^
Pt–Al_2_O_3_		1.600	400	Kim et al.^70^
NiFe/CeAl		0.513	400	Yang et al.^71^
NiCr/CeAl		0.454	400	Yang et al.^71^
Ni/CeAl		0.461	400	Yang et al.^71^
Ni/Al		0.469	400	Yang et al.^71^
Rh/S-1		0.333	300	Wang et al.^72^
Rh@S-1		0.167	300	Wang et al.^72^

### 3.7 Stability and Switchability Test

The stability of the catalysts is an essential parameter to evaluate its potential for use in large-scale applications. Because it has been widely described in the literature about the rapid sintering and deactivation of Ni-based catalysts in the CO_2_ methanation reaction and especially in RWGS,^73^ we evaluated the stability of the Cs–Ni/CeO_2_ catalyst, which presented the most relevant performance in terms of selectivity in the low-temperature RWGS reaction. This catalyst presented the best compromise between higher CO selectivity and CO_2_ conversion. The stability tests were conducted over four cycles of 24 h switching from 350 to 700 °C in order to mimic a change between low-temperature RWGS conditions and the commonly used RWGS reaction conditions.

**Figure 10:**
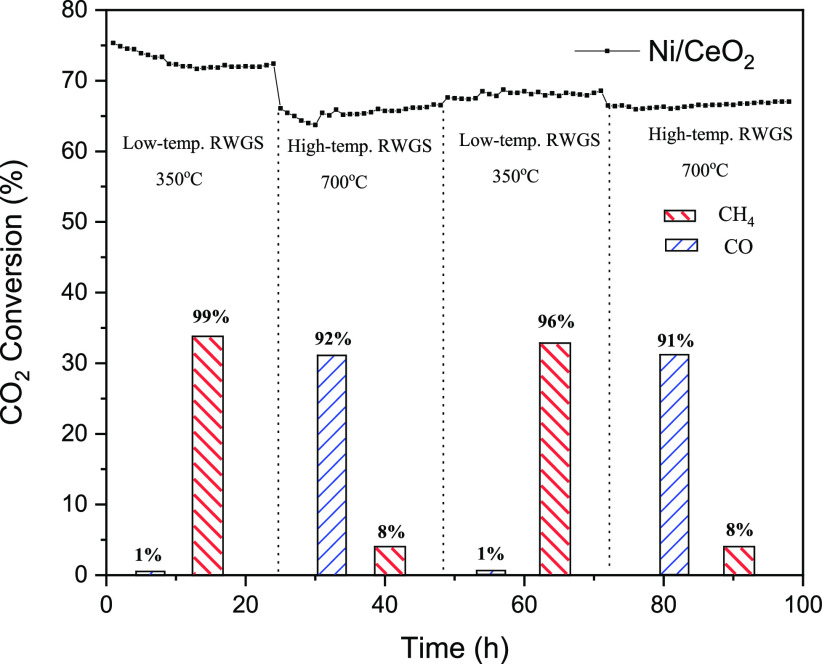
Stability test for the nonreduced Ni/CeO_2_ catalyst, switching temperature from 350 to 700 °C. CO_2_ conversion is shown as dotted lines, CH_4_ selectivity as red bars, and CO selectivity as blue bars. Conditions: atmospheric pressure, WHSV = 12,000 mL g_cat_^–1^ h^–1^, H_2_/CO_2_ = 4:1.

For the sake of comparison with the reference system, Figure 10 shows the stability test in Ni/CeO_2_ varying the temperature from 350 to 700 °C, where the track of CO_2_ selectivity is combined over the analysis time and the associated product selectivity (shown as bars due to the stability of the catalyst through the step). The results clearly show that Ni/CeO_2_ is stable in each reaction range, just showing a slight catalytic activity decrease after the first cycle. However, especially in the RWGS cycles, a decrease of ca. 10% is observed in the catalytic activity, possibly attributed to the partial sintering of Ni particles. The switch in the cycle highly affects the product selectivity, which shows the successful transition from the low-temperature RWGS to high-temperature RWGS. For instance, the selectivity products perfectly match with the values of the catalytic activity described in Figure 7.

**Figure 11:**
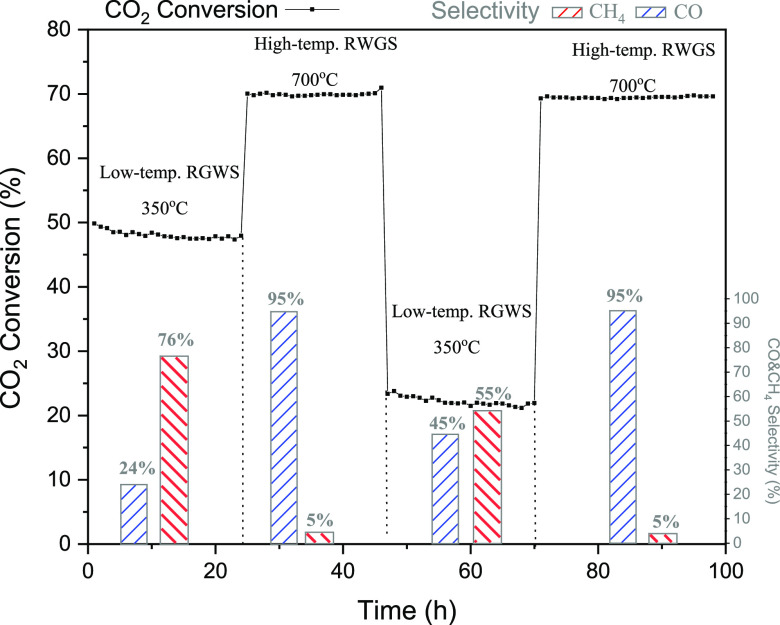
Stability test for the nonreduced Cs–Ni/CeO_2_ catalyst, switching temperature from 350 to 700 °C. CO_2_ conversion is shown as the dotted line, CH_4_ selectivity as red bars, and CO selectivity as blue bars. Conditions: atmospheric pressure, WHSV = 12,000 mL g_cat_^–1^ h^–1^, H_2_/CO_2_ = 4:1.

Figure 11 shows the evolution of CO_2_ conversion, and its product selectivity through the four cycles switching from low-temperature (350 °C) to high-temperature (700 °C) RWGS reaction conditions in the Cs-promoted Ni/CeO_2_ catalyst (Cs–Ni/CeO_2_). In the first cycle, the catalyst was reduced in situ, showing similar values of CO_2_ conversion and product selectivity, as reported above. Additionally, at 350 °C, the CO selectivity improved up to 24% compared to that of the monometallic catalyst, and the activity remained stable for 24 h. The second step in cycle 1 corresponds to the RWGS reaction conditions, where not only a higher CO_2_ conversion was observed but also the CO selectivity remained stable for up to 24 h. However, in cycle 2, which corresponds to the catalyst already reduced during the reaction, the catalytic activity suffered from a decrease in the CO_2_ methanation conditions due to the sintering of the particles, as was observed in Figure 7. Despite the decrease in the catalytic activity, under these conditions, the methanation suffered a remarkable suppression, and the CO selectivity was boosted up to 45% compared to that of the monometallic catalyst. Finally, in the second step of cycle 2, the catalyst was subject to a second time to the RWGS conditions (700 °C), showing a similar CO selectivity as in cycle 1 again and giving evidence of its high stability after almost 100 h of experiment. This reflects the effect of Cs on not only boosting the CO selectivity but also the stability on the catalyst via Ni particle stabilization, which makes this catalyst a potential candidate for RWGS. It is highly advantageous in terms of energy optimization to have a catalyst that presents a CO selectivity up to 45% at low temperatures (350 °C) rather than at 600 °C as required for the Ni/CeO_2_ catalyst.

### 3.8 Characterization of the Spent Catalysts

**Figure 12:**
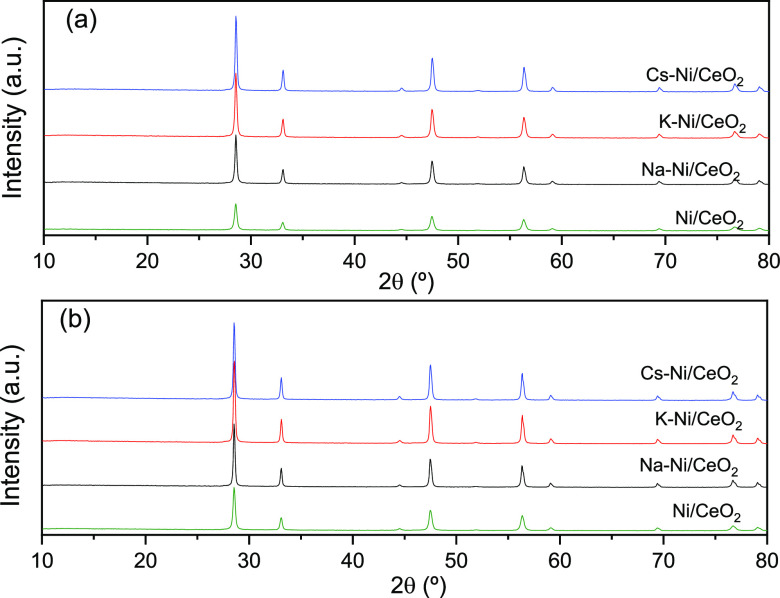
XRD patterns of spent (a) nonreduced and (b) reduced catalysts.

The XRD patterns of the spent catalyst provide valuable information related to the effect of the promoters on the active phase of the catalyst after the catalytic performance. In addition to the stability tests described in the previous section for both the nonpromoted and Cs-promoted catalysts, Figure 12 shows the XRD patterns of the spent catalyst, both nonreduced and reduced. From these XRD patterns, an increase in the Ni particle size was observed after the incorporation of promoters (Table 1); whereas from Table 5 which summarizes the Ni particle size of the spent catalysts, a considerable increase in the Ni particle size was observed in the nonpromoted catalyst, mainly attributing to the widely described sintering effect of Ni. However, the average particle size of the spent promoted catalysts remained more stable, which give evidence of the effect of alkali promoters in the sintering of Ni nanoparticles.

**Table 5:** Average Crystal Size of Ni in Spent Catalysts

	crystal size (nm)	
	Spent	
catalyst	Ni	Ni (reduced)
Ni/CeO_2_	28.3	29.6
Na–Ni/CeO_2_	38.2	39.5
K–Ni/CeO_2_	29.7	30.1
Cs–Ni/CeO_2_	28.3	28.7

## 4 Conclusions

The addition of alkali metals in Ni-based catalyst systems was evaluated as a strategy in the design of novel flexible catalysts for the RWGS reaction in both low- and high-temperature ranges, facilitating a potential coupling between FT or methanol conversion and RWGS reactions. It was observed that the addition of Na, Cs, and K into the Ni/CeO_2_ catalyst modified not only the textural properties of the catalyst but also the chemical and electronic surface properties as was observed from the XPS and H_2_-TPR measurements, which has an inherent effect on the catalytic performance. For instance, it was observed that the addition of alkali metals improved the reducibility and also the dispersion of the active phase of the Ni/CeO_2_ catalyst. The catalytic activity was studied on both the nonreduced and H_2_ pre-reduced catalysts and the effect of pretreatment conditions on the selectivity of the products was observed. Furthermore, the boosting effect of the alkali metals on the CO selectivity was observed, especially in the Cs–Ni/CeO_2_ catalyst, with an increase of ca. 40% in CO selectivity at 350 °C compared to the nonpromoted catalysts. Additionally, the stability test performed in the Cs–Ni/CeO_2_ catalyst showed that this catalyst could switch from low-to high-RGWS reaction, obtaining a relevant performance in terms of CO selectivity compared with that of the Ni/CeO_2_ catalyst. We acknowledge that further research is needed to improve the catalyst’s activity in low-temperature ranges and to fully suppress methanation. In this sense, our multi-component catalysts open new research avenues toward the design of switchable low/high-temperature RWGS reaction units for gas-phase CO_2_ conversion, representing a step ahead in catalyst optimization to combat global warming.

## Supporting Information Available

Pore size distribution of the evaluated catalysts calculated using the BJH model (PDF)
