# Supplementary material for: Design of Full-Temperature-Range RWGS Catalysts: Impact of Alkali Promoters on Ni/CeO2
Source: Energy Fuels. 2022 May 25;36(12):6362–73. doi: 10.1021/acs.energyfuels.2c00784 (PMC9945166; doi:10.1021/acs.energyfuels.2c00784)
Supplement: Supplementary file 1 — ef2c00784_si_001.pdf [file ef2c00784_si_001.pdf]

SUPPORTING INFORMATION

Design of Full-Temperature-Range RWGS Catalysts: Impact of Alkali Promoters on Ni/CeO_2

Jesus Gandara-Loe [a*], Qi Zhang [b], Juan José Villora-Picó [c], Antonio Sepúlveda-Escribano [c], Laura Pastor-Pérez [a,b], Tomas Ramirez Reina [a,b]

[a] *Department of Inorganic Chemistry and Materials Sciences Institute, University of Seville-CSIC, 41092, Seville, Spain*

[b] *Department of Chemical and Process Engineering, University of Surrey, Guildford, GU2 7XH, United Kingdom*

[c] *Laboratorio de Materiales Avanzados, Departamento de Química Inorgánica -Instituto Universitario de Materiales de Alicante, Universidad de Alicante, Alicante, E-03080, Spain*

* Corresponding author:

jgloe@us.es *(Jesus Gandara-Loe)*

TABLE OF CONTENT

Figure S1……………………………………………………………………………………………S-3

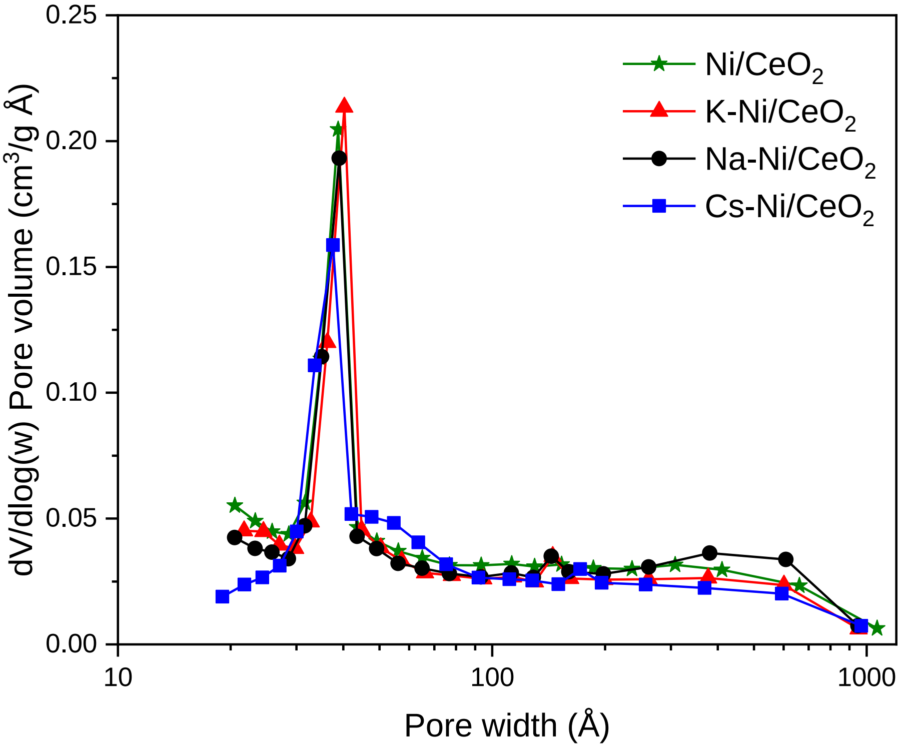

Figure S1. Pore size distribution of the catalyst calculated on the desorption branch using the BJH model.
